# Integrating Advanced Technologies for Environmental Valuation in Legacy Mining Sites: The Role of Digital Twins at Lavrion Technological and Cultural Park

**DOI:** 10.3390/s25195941

**Published:** 2025-09-23

**Authors:** Miguel Ángel Maté-González, Cristina Sáez Blázquez, Sergio Alejandro Camargo Vargas, Fernando Peral Fernández, Daniel Herranz Herranz, Enrique González González, Vasileios Protonotarios, Diego González-Aguilera

**Affiliations:** 1 Cartographic and Land Engineering Department, Higher Polytechnic School of Avila, Universidad de Salamanca, Hornos Caleros 50, 05003 Ávila, Spain; u107596@usal.es (C.S.B.); sacamargov@usal.es (S.A.C.V.); fernandopf@usal.es (F.P.F.); danielherranz@usal.es (D.H.H.); egonzalezgonzalez@usal.es (E.G.G.); daguilera@usal.es (D.G.-A.); 2 Lavrion Technological and Cultural Park, 19500 Lavrion, Greece; btproto@yahoo.com

**Keywords:** industrial cultural heritage, mining environments, Geology, environmental pollution, environmental management, sustainability, Digital Twin, Internet of Things (IoT), virtual tour, 3D modeling, Geophysics, technological innovation, digital transformation, heritage recovery and conservation

## Abstract

**Highlights:**

**What are the main findings?**

- Development of a Digital Twin for a waste storage deposit in a mining context.
- Integration of real-time sensor monitoring, geophysical methods, and 3D modeling.

**What is the implication of the main finding?**

- Continuous tracking of critical parameters for proactive risk management.
- Evaluation of hazardous-waste storage, structure conditions, and contaminated soils.

**Abstract:**

The rehabilitation of mining environments poses significant challenges due to the contamination risks associated with hazardous materials, such as arsenic and other chemical products. This research study presents the development of a Digital Twin for the Lavrion Technological and Cultural Park (LTCP), a former mining and metalworking site that is currently undergoing environmental restoration. The Digital Twin integrates advanced technologies, including real-time sensor monitoring, geophysical methods, and 3D modeling, to provide a comprehensive tool for assessing and managing the environmental conditions of the site. Key elements of the project include the monitoring of hazardous-waste storage, the evaluation of contaminated soils, and the assessment of the Park’s infrastructure, which includes both deteriorating buildings and successfully restored structures. Real-time sensor data are collected to track critical parameters such as conductivity, temperature, salinity, and levels of pollutants, enabling proactive environmental management and mitigation of potential risks. The integration of these technologies enables continuous monitoring, historical data analysis, and improved decision making in the ongoing efforts to preserve the site’s ecological integrity. This study demonstrates the potential of using Digital Twins as an innovative solution for the sustainable management and valorization of mining heritage sites, offering insights into both technological applications and environmental conservation practices.

## 1. Introduction

Throughout history, humankind has relied on subsoil resources as fundamental components for its development and technological advancement. Since the dawn of civilization, raw materials have played a crucial role in the manufacture of tools, the creation of materials, and the development of technologies that have driven human progress [1,2]. Historically, mineral resources have been essential to infrastructure development, metallurgy, and the production of ornamental artifacts symbolizing power. Currently, these raw materials are indispensable across diverse high-technology sectors, including mechanical engineering, biomedical applications, food industry additives, and advanced technologies such as electronics (semiconductors, batteries, etc.), telecommunications (optical fibers, 5G components, etc.), and renewable energy systems, as well as emerging fields like artificial intelligence, robotics, and quantum computing [3].

Beyond their essential role in manufacturing goods and services, raw materials extracted from the subsurface underpin the global economy, propel industrial development, and contribute significantly to enhancing quality of life. Their extraction and use have been decisive in the transformation of societies, shaping their economic, technological, and social structures [4]. In this context, these resources have acquired a strategic character not only to face global challenges such as energy transition, climate change, and technological innovation but also as a key tool to consolidate and demonstrate the geopolitical power of nations [5,6,7].

Despite the above, the use of subsoil resources faces significant challenges, especially in the mining context. Mining activities, which are essential to the extraction of raw materials, are usually associated with significant impacts on the environment that require rigorous management [8,9]. During the extraction and processing processes, large quantities of solid and liquid waste, as well as atmospheric emissions, are produced. If these are not properly managed, they can cause serious pollution problems affecting soil, water bodies, and air quality. Among the most problematic pollutants associated with these activities are heavy metals such as arsenic, mercury, and lead, as well as highly toxic chemicals such as cyanide, used in extraction processes such as leaching. These pollutants not only have a direct impact on ecosystems but also represent a serious danger to human health, as they can cause chronic diseases, poisoning, and in extreme cases, irreversible effects on exposed populations [10,11]. Of particular concern is the persistence of these pollutants in the environment. Both heavy metals and chemical compounds can remain active for decades or even centuries, affecting biodiversity and altering ecosystems for a long time. This contamination has cumulative effects, as polluted soils and degraded aquifers not only hinder the development of agricultural or livestock activities but also pose a constant threat to nearby human communities and the environment [12,13].

In this context, abandoned or inactive mining facilities often remain sources of environmental contamination due to inadequate decommissioning, posing significant risks to ecosystems and human health. Consequently, the rehabilitation of former mining sites is a critical priority aimed at mitigating environmental damage, restoring ecosystems, and enabling sustainable land use. Beyond environmental concerns, these initiatives also promote the cultural revaluation of historic mines as industrial heritage, recognizing their historical importance in economic development, technological innovation, and community identity [14,15]. Ensuring contaminant control and site safety is essential to balancing heritage preservation with environmental and public health protection [16,17,18,19].

Post-mining areas are also prone to emergency situations such as sudden ground subsidence, slope failures, or uncontrolled release of Acid Mine Drainage (AMD). These events have been widely documented in Europe and worldwide, causing damage to infrastructures and contamination of aquifers and putting local populations at risk [20,21]. The inadequate sealing of shafts and galleries, together with the progressive deterioration of isolation barriers, often leads to delayed failures that can occur decades after mine closure. Therefore, the inclusion of systematic monitoring and risk assessment strategies is crucial to mitigating such incidents and ensuring the long-term safety of these areas.

Different strategies have been proposed to minimize the environmental impact of mine closure, including proper backfilling, installation of multilayer geotechnical barriers, leachate drainage systems, revegetation of disturbed soils, and long-term hydrogeological monitoring [22]. In parallel, the reuse of mining infrastructure for scientific, educational, or cultural purposes has been successfully implemented in several countries, transforming former mining sites into museums, technology parks, geoparks, or training centers [23,24]. These complementary approaches contribute to both environmental protection and socio-economic revitalization of the affected regions.

For all these reasons, the management of contaminated sites represents a complex challenge that requires a multidisciplinary approach, combining traditional environmental remediation methods with innovative technologies to improve the effectiveness of monitoring, assessment, and remediation. In recent years, advanced approaches that enable the continuous monitoring of environmental conditions, identification of contamination hotspots, and efficient implementation of corrective measures have been developed. The use of technologies such as real-time monitoring sensors, Geographic Information Systems (GISs), geophysical techniques, satellite imagery, and geometric modeling enable continuous environmental monitoring, precise identification of contamination hotspots, and efficient remediation [25,26]. These technologies provide accurate, up-to-date data; support informed decision making; and facilitate proactive pollution management by anticipating potential future issues [27,28,29].

In this sense, Digital Twins (DTs) represent a cutting-edge tool that combines real-time sensor data with precise geometric or descriptive models to create dynamic virtual replicas of physical sites or systems. These models enable simulation of evolving conditions, risk assessment, and the efficient planning of interventions with reduced need for invasive actions. By leveraging remote sensors and cloud platforms, DTs enhance data accessibility, coordination, and responsiveness. In industrial applications, they optimize costs, streamline maintenance, improve production efficiency, and support proactive failure detection, fostering sustainable management. Digital Twins are currently applied across diverse sectors, also including the mining context, where they are used as a tool for increasing underground knowledge and facilitate the planning of restoration and management actions [30,31].

For the development of a DT, advanced models such as Building Information Modeling (BIM), Computational Fluid Dynamics (CFD), Finite Element Analysis (FEA), and Computer-Aided Design (CAD) can be used [32]. These models, under the umbrella of an integrated platform and real-time information through the Internet of Things (IoT), make it possible to develop highly detailed and accurate representations of complex infrastructures and systems, such as buildings, underground facilities, bridges, or industrial plants [33]. These representations not only serve to model and analyze structural and mechanical aspects but also enable damage identification, simulation of operating conditions, and the efficient planning of interventions. In addition, they integrate capabilities to optimize critical processes such as predictive maintenance, lifecycle management, and risk assessment, providing solutions that increase operational efficiency, minimize costs, and reduce downtime. This approach facilitates a deeper understanding of the system behavior and more informed and proactive decision making, which is an essential value in the mining sector [34,35,36].

In dynamic environments, Digital Twins incorporate IoT sensors, external real-time data (e.g., traffic and weather), and updated geospatial information processed through GISs [37]. This integration supports the management of complex ecosystems such as mining operations and large-scale projects by providing accurate, real-time insights that improve strategic planning, risk mitigation, sustainability, and system resilience. On the other hand, interactive Digital Twins, based on virtual reality (VR) technologies, offer immersive experiences and advanced monitoring. These DTs enable highly realistic virtual tours of physical environments, combining interactive exploration with real-time visualization of data from IoT sensors. This approach is particularly valuable for the conservation, management, and condition monitoring of elements or objects, as it enables to generate early warnings, prevent damage through constant monitoring, and anticipate possible future scenarios through predictive techniques. Consequently, it achieves a balance between virtual accessibility and physical preservation, promoting sustainable mining practices, improved asset protection, and more efficient operational management [38,39].

Based on the opportunities of the mentioned technologies, this research study presents the development of an experimental Digital Twin of the Lavrion Technological and Cultural Park (LTCP) using virtual reality tools. The Digital Twin accurately replicates a key area undergoing restoration, providing an advanced platform for management, monitoring, and analysis. The work details each development stage, from IoT sensor data collection to geometric and environmental characterization, culminating in an interactive, user-friendly digital platform. This work demonstrates how Digital Twins can revolutionize environmental management in mining contexts by integrating sustainability, technology, and heritage conservation while fostering cultural valorization and comprehensive solutions to technical and environmental challenges.

The paper is organized as follows: The following section details the components, techniques, tools, and data used to develop the experimental Digital Twin. The Results Section provides the historical and cultural background of the LTCP, focusing on the characterization of a sealed hazardous-waste landfill selected as a case study for platform validation. It also presents the integration of the collected data into a comprehensive model supporting the Digital Twin and highlights its monitoring and environmental management capabilities. Finally, the Discussion and Conclusions Sections analyze the main findings, evaluate the Digital Twin’s impact and practical applications, and suggest potential improvements and future directions for managing similar environments.

## 2. Materials and Methods

As mentioned in the previous section, this work details the creation of an experimental interactive Digital Twin, developed using advanced virtual reality technologies. This tool not only seeks to offer an immersive experience but also aims to act as an innovative way for environmental monitoring and management, along with the possibility of performing a cultural valorization of the environment under restoration.

**Figure 1:**
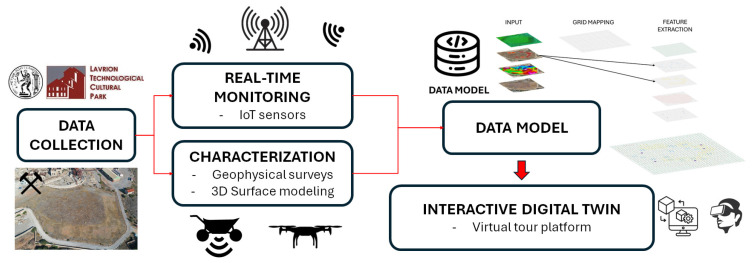
Outline of the methodology used for the creation of the DT platform.

To this end, extensive data collection was carried out on the case under study, integrating a set of advanced technologies to ensure a comprehensive understanding of the analysis environment (Figure 1). First, a real-time monitoring system based on IoT sensors was implemented, designed to continuously collect and analyze key parameters of the site. In addition, a detailed characterization of the reservoir, both inside and outside, was carried out. In this sense, advanced geophysical surveys were applied to identify vulnerable points and key structural features. Three-dimensional (3D) modeling techniques were also used to generate an accurate representation of the working environment.

Based on the above information, together with additional data available from the landfill, a comprehensive data model was developed. This model provides a complete understanding of the environmental and structural context of the landfill, combining the analysis of physical and chemical characteristics with continuous monitoring based on IoT sensors. This approach seeks to detect possible anomalies early, generate preventive alerts, and facilitate rapid and informed decision making, thus optimizing the management and preservation of the environment.

This data model was integrated into a Digital Twin interactive platform, designed to offer an immersive virtual experience. Through a virtual tour, it seeks to facilitate both the exploration of the environment and the visualization and analysis of data in real time, providing the possibility of interacting dynamically with the information collected and processed.

### 2.1. Study Case: Lavrion Technological Cultural Park (LTPC)

**Figure 2:**
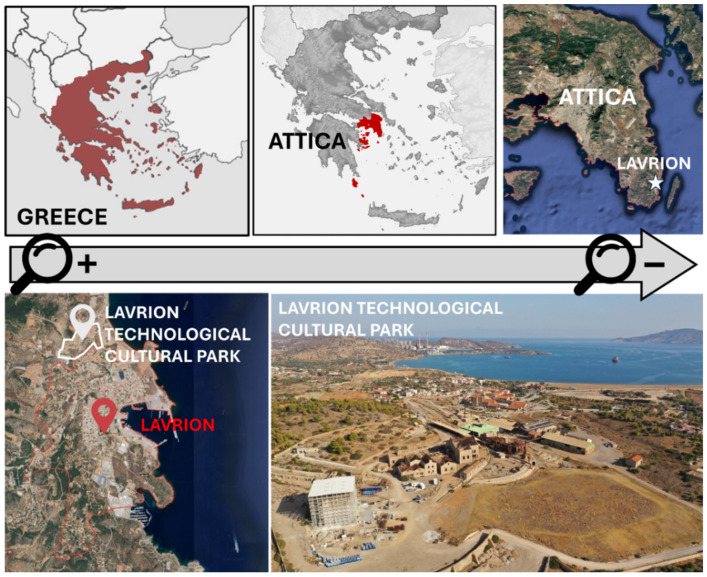
Location and general view of the Lavrion Technological and Cultural Park (LTCP).

As already mentioned, this research study is focused on the Lavrion Technological and Cultural Park (LTCP). Lavrion is a coastal town in southeastern Attica, Greece. It is located about 60 km southeast of Athens, on the shores of the Aegean Sea. The area is characterized by a warm semi-arid climate, with mild winters and hot summers. Average summer temperatures reach around 30 °C, with August being the hottest month (highs around 30 °C and lows around 24 °C). The rainy season lasts from October to April, with December being the wettest month. Regarding its population, currently comprising around 7500 residents, it has historically been shaped by its mining industry. In ancient times, the silver mines attracted workers and traders, forming a vibrant community that contributed to Athens’ wealth. Today, the town’s residents continue to preserve this heritage, with cultural events, museums, and the LTCP celebrating the city’s mining legacy while promoting education, technology, and sustainable development. This Park is an initiative of the National Technical University of Athens (NTUA) aimed at fostering innovation and culture in a region that was once an epicenter of industrial development in Greece. Founded in 1992 on the premises of the former French Mining Company of Lavrion, the LTCP presents itself as a collaborative space designed to promote the generation of innovative ideas and solutions while preserving and enhancing the valuable historical and industrial heritage of the area. The LTCP is located in Lavrion, a port city known for its rich mining history, in the southeastern part of the Attica region of Greece. Below, Figure 2 shows the location and general view of the LTCP.

**Figure 3:**
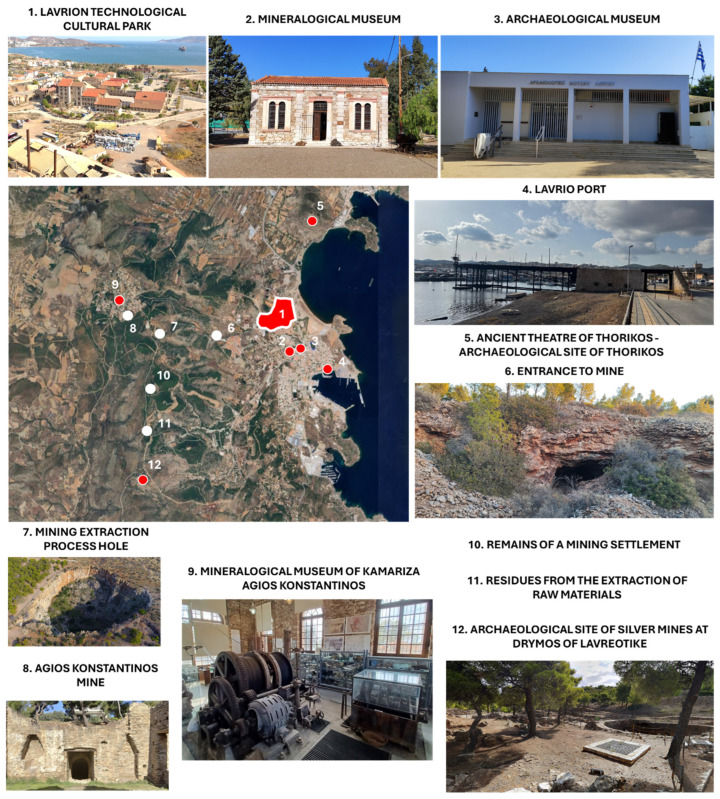
Different vestiges of mining activities in the Lavrion Technological Cultural Park area. In red are those elements of great cultural and touristic interest which can be visited.

Nowadays, the LTCP covers 60.78 hectares and includes 41 restored buildings that blend historical value with modern functionality, housing companies, NTUA laboratories, and cultural and educational institutions. Lavrion has a long mining tradition dating back before 3000 BC, notably silver mining, which financed Athens’ rise to power [40]. In the 19th century, the area reemerged as an industrial hub thanks to French Mining Company of Lavrion, driving economic development, shaping the local urban identity, and consolidating Lavrion as a reference in the country’s mining and metallurgical industry [41,42,43]. As an example of the mentioned mining activity, Figure 3 presents some of the main vestiges in the LTCP area.

However, during the 1970s and 1980s, a crisis hit the main Greek industrial centers, including Lavrion, leading to the closure of industrial plants and an increase in unemployment in the region [44]. In response, the NTUA, in collaboration with the local community, proposed the reuse of industrial facilities to create the LTCP, with the aim of revitalizing the area through research, educational, and cultural activities. In this sense, environmental recovery has been one of the biggest challenges for the Park, due to the serious pollution problems caused by centuries of mining and metallurgical activity. With the aim of addressing this situation, the following initiatives have been developed to transform the area into a safer and more sustainable environment:

- Excavation and management of contaminated soils: Heavily contaminated soils with heavy metals and hazardous waste were removed and transferred to a specially designed landfill within the Park, following strict environmental regulations. Clean materials were then used to refill the areas, enabling their safe recovery.
- Underground storage of highly hazardous waste: A specially designed underground facility was built for the safe long-term storage of highly toxic waste, such as arsenic compounds. Waste is sealed in specialized containers to prevent leaks or contamination.
- Establishment of an environmental monitoring laboratory: A dedicated laboratory was set up to continuously monitor soil, water, and air quality, ensuring regulatory compliance and supporting the safe operation of remediation projects and facility maintenance.
- Rehabilitation of LTCP historic buildings: Historic buildings within the LTCP, heavily contaminated by past industrial activity, are being restored. A key example is the “Konofagos” building, undergoing thorough decontamination and rehabilitation in line with EU and Greek regulations. The restored site will serve new cultural and innovative purposes while preserving its historical legacy.

Today, environmental monitoring and control still represent a fundamental axis in the Park’s management, with the purpose of supervising and analyzing all potentially dangerous elements of the area. Due to advances in environmental recovery, the LTCP has established itself as a model of sustainable development, combining the preservation of historical heritage with the creation of new technological, educational, and cultural opportunities. This transformation has revitalized the local community and demonstrated how the reuse of industrial facilities can be successfully harmonized with environmental protection, serving as a reference for similar projects in the future [45].

#### Hazardous-Waste Landfill

**Figure 4:**
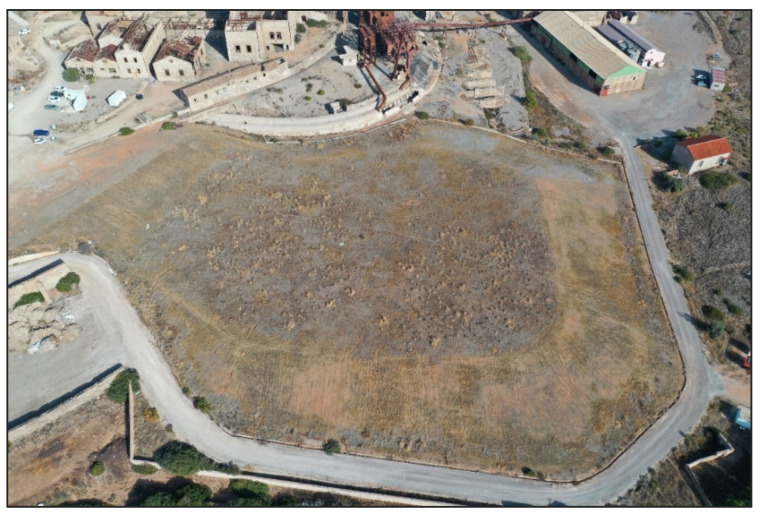
Hazardous-waste landfill located at the LTCP.

The site under study corresponds to a hazardous-waste deposit (known as “Dry Tomb”) derived from mining activities, located in an area of environmental and strategic interest due to its proximity to protected natural areas and inhabited areas. Its origin dates back to the last decades of the 20th century, when it was used as a controlled deposit to house toxic byproducts generated in the extraction and processing of metallic minerals (Figure 4). During its exploitation, the site received mainly residues rich in heavy metals, such as lead, zinc, arsenic, manganese, and cadmium, which are highly contaminating and have high environmental persistence.

The landfill is structured as an engineered deposit, created by layering contaminated materials separated by technical isolation barriers such as geotextile sheets and internal drainage systems. Over time, however, the integrity of these barriers has started to decline, raising increasing concerns about potential environmental impacts like leachate leakage, contamination of soil and groundwater, and release of harmful gases.

The mentioned deposit is currently out of operation but still requires active monitoring and restoration measures. The complexity of its internal structure, the heterogeneity of the deposited materials, and the lack of detailed knowledge of the current confinement conditions make it necessary to resort to advanced non-invasive methodologies for its characterization. In this context, geophysical techniques, environmental sensing, and immersive technologies have been used to develop an integrated diagnostic and management system, with the aim of guaranteeing environmental safety and facilitating its restoration and future valorization [46,47].

### 2.2. Deposit Characterization

#### 2.2.1. IoT Monitoring Systems

The use of LoRaWAN (Long-Range Wide-Area Network)-based IoT monitoring was preferred over short-range wireless protocols such as Wi-Fi or ZigBee due to its superior communication range, low power consumption, and ability to operate reliably in semi-rural areas with limited network infrastructure. LoRaWAN technology enables coverage of the entire landfill site with a single gateway, minimizing installation costs and maintenance requirements. This approach enables near-real-time transmission of environmental parameters (soil moisture, conductivity, NPK concentrations, etc.) without the need for permanent power lines or wired data connections.

For the implementation of an efficient environmental monitoring system, it is essential to select a robust communication architecture suitable for the needs of the environment. In this case, a network based on LoRaWAN technology, a communication protocol designed for data transmission over long distances with reduced energy consumption, was chosen. This type of network is particularly useful in scenarios where traditional connectivity, such as Wi-Fi or mobile networks, is not viable due to infrastructure restrictions, energy consumption, or operating costs. LoRaWAN is a low-power, long-range wireless technology that enables sensor data transmission over large areas without wiring. It offers up to 15 km range in open spaces, low energy use for years-long battery life, strong security, high scalability, and easy cloud integration [48].

**Figure 5:**
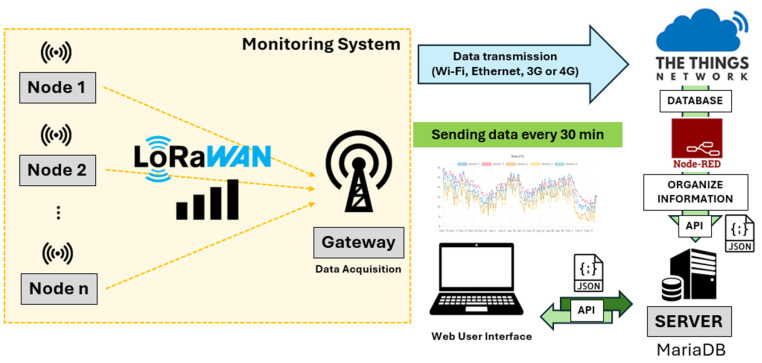
Schematic diagram of the methodology followed in the configuration of the monitoring system.

In the case of this research study, the monitoring system implemented is based on a typical LoRaWAN network configuration, composed of three fundamental elements:

- First, LoRaWAN IoT sensors, which are devices responsible for measuring different environmental variables that vary depending on the parameters recorded. Their placement is planned to ensure optimal coverage and efficient data collection. These sensors use minimal energy, allowing long autonomous operation and periodic data transmission.
- Secondly, the LoRaWAN gateway, which acts as a bridge between sensors and data infrastructure. It receives signals from sensors, processes them, and forwards the data via internet (Ethernet, Wi-Fi, or mobile) to a central server. One gateway can connect multiple sensors, reducing infrastructure complexity.
- Finally, the collected data are stored on the cloud platform The Things Network (TTN). Data are processed by NODE-RED, which receives it from TTN via MQTT and sends it to a web server through an API in JSON format. The API supports tools for visualization, analysis, anomaly detection, and decision making. This setup is efficient for remote areas, has low cost, and enables real-time data delivery with minimal infrastructure. The general diagram followed for the configuration of the monitoring system is presented in Figure 5. In addition, Table 1 includes the main characteristics of LoRaWAN technology.

Regarding the sensors, the NPK-7in1 LoRaWAN Soil NPK Sensor EU868, developed by Dragino, Shenzhen, China, (hereafter the NPK-7in1 sensor), a device designed for the accurate measurement of key soil parameters, was chosen in this work. This sensor is composed of two fundamental parts that work together to ensure efficient, long-range monitoring.

The first part is the measurement probe, responsible for obtaining essential soil data, including nitrogen (N), phosphorus (P), potassium (K), moisture, temperature, electrical conductivity, and salinity, using the Time-Domain Reflectometry (TDR) technique. Designed for outdoor applications, the probe is IP68-certified, allowing it to be permanently buried and operate under adverse environmental conditions, ensuring continuous and reliable monitoring over time.

The second part is the processing and communication unit, equipped with an 8500 mAh Li-SOCI2 battery, which provides an autonomy of up to five years, minimizing the need for frequent maintenance. This unit not only processes the data captured by the probe but also transmits the information using LoRaWAN technology, allowing communication over long distances with minimal power consumption. Thanks to this spread-spectrum technology, the sensor offers high immunity to interference, ensuring the reliability of transmitted data even in environments with possible electromagnetic interference [49]. The connection between the measuring probe and the processing and communication unit is made via a rugged cable, designed to efficiently transmit the collected data without signal degradation.

In addition, each NPK-7in1 sensor comes preloaded with a unique set of keys for registration with the LoRaWAN server, allowing automatic connection upon activation. Below, Table 2 includes the main characteristics of the NPK sensors implemented in the present research study.

Despite their advantages, IoT sensors are subject to several limitations that must be considered. Signal attenuation can occur due to vegetation, topographic obstacles, or electromagnetic interference, potentially causing temporary data loss. Battery life is finite and can be reduced by extreme temperatures, requiring periodic replacement or recharging. Additionally, sensors may require regular calibration to avoid drift in measured parameters, particularly for conductivity and salinity measurements. Data transmission through LoRaWAN can also be affected by gateway failures or network saturation in high-density deployment. To mitigate these issues, a redundant sensor deployment strategy, periodic maintenance schedule, and backup communication protocols were implemented in this study.

**Figure 6:**
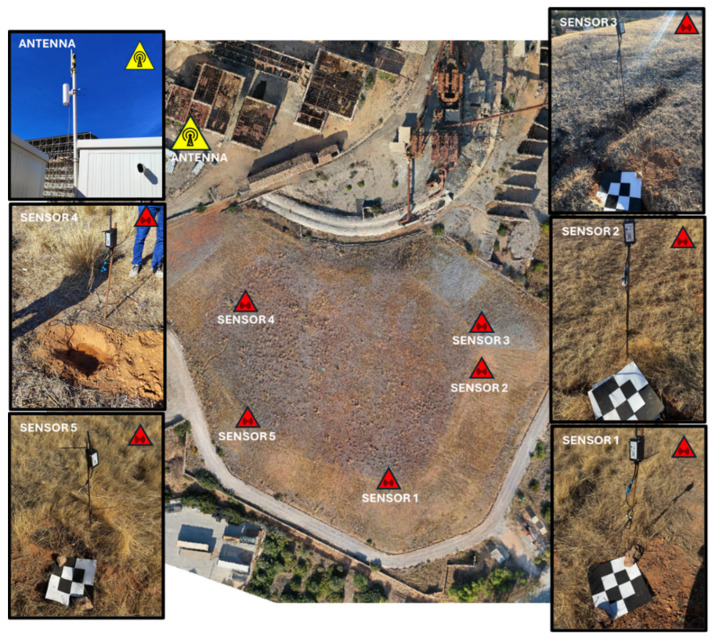
Sensor distribution and gateway location.

**Figure 7:**
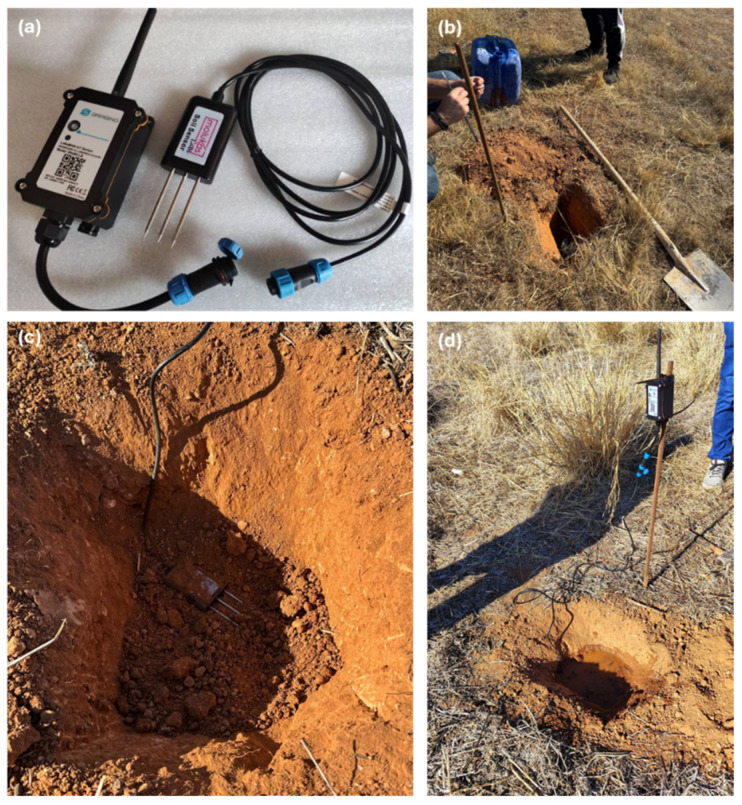
(**a**) NPK-7in1 LoRaWAN Soil NPK Sensor EU868. (**b**) Excavation of the hole in the soil and placement of the steel rod for the processing unit. (**c**) Installation of the sensor in horizontal position inside the hole. (**d**) Covering of the sensor with soil and controlled watering to compact the soil and ensure optimal contact with the substrate.

For site monitoring, five sensors were strategically placed within the landfill, targeting areas identified by prior geophysical surveys as having complex material distributions or zones of particular interest for evaluation (Figure 6). For the installation of the sensors, a hole was dug in the ground at a depth of 30 cm in which the devices were placed horizontally to ensure optimal measurement of soil properties. After placement, the sensors were covered again with soil, and controlled irrigation was performed in order to compact the soil and ensure adequate contact with the substrate (Figure 7). Additionally, the battery module, which also acts as a signal transmitter, was installed on top of a steel rod. This arrangement improves the coverage of the LoRaWAN network, optimizing data transmission from the sensors to the gateway.

As can be observed below in Figure 6, each sensor was marked with a high-visibility target, designed to be clearly identifiable from the air during the photogrammetric flight. This allows the sensors to appear accurately in the generated orthophotography, facilitating their geolocation and ensuring their exact identification in the final data model.

Regarding the gateway, a Dragino DLOS8N Outdoor LoRaWAN Gateway with 4G (hereafter, the Dragino DLOS8N Gateway) was selected, serving as a high-performance communication bridge between the LoRaWAN sensors and the data network. It runs on an open-source embedded Linux system with flexible management options via GUI or SSH. Compatible with LoRaWAN protocols and multiple-packet forwarders, it supports diverse communication infrastructures. The device offers Wi-Fi, Ethernet, and 3G/4G connectivity for reliable data transmission in remote areas and features robust hardware with IP65 protection and lightning safeguards, which make it suitable for outdoor use [50].

**Figure 8:**
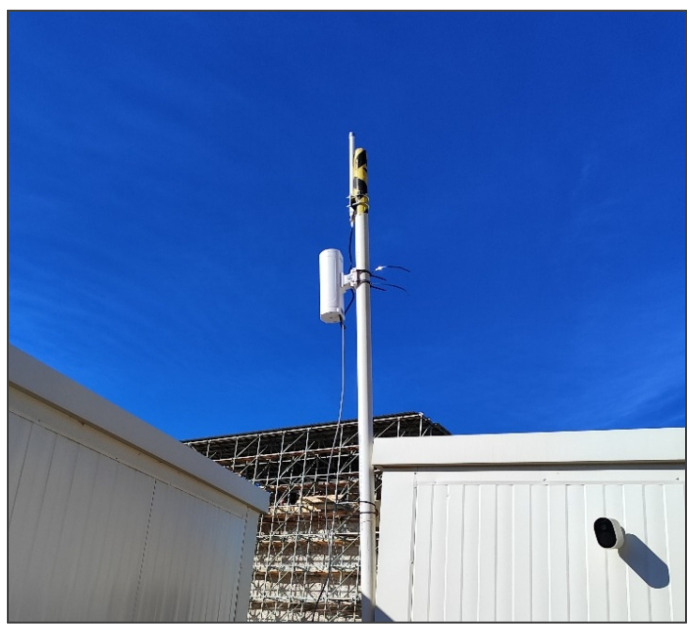
Placement of the Dragino DLOS8N Gateway.

It is worth to mention that the Dragino gateway was installed in an elevated area (Figure 8), specifically on an antenna mast located in a site hut with Ethernet connection and direct visibility over the landfill. This location ensures optimal coverage of the LoRaWAN network, ensuring efficient communication with all sensors deployed in the area. Additionally, a 5G mobile data SIM card was incorporated as a backup in case of loss of the Ethernet connection, allowing the system to continue sending and collecting data without interruption.

#### 2.2.2. Exterior 3D Model Using Geomatics Techniques

There are several techniques and methodologies for the geometric documentation of scenarios, each with specific characteristics that make them more suitable according to the context and requirements of the project. Among the most prominent are photogrammetric techniques and laser techniques, both widely used in the field of geomatics to obtain high-precision three-dimensional models.

Laser techniques or LiDAR (Light Detection and Ranging) technology, in turn, can be divided into two broad categories: terrestrial laser scanning (TLS), which is based on data capture using static scanners positioned at strategic points, and Wearable Mobile Mapping Systems (WMMSs), which enable dynamic data acquisition through portable devices or devices mounted on moving platforms. The latter methodology offers significant advantages in terms of speed and coverage, especially in complex or difficult-to-access environments [51,52].

In this work, after assessing available techniques, a combination of methods was chosen to optimize the geometric documentation of the study area. For general documentation, aerial photogrammetry was selected, enabling the construction of high-precision 3D models from drone-captured images. Besides 3D models, this method produces orthophotographs, useful for detailed morphological and environmental evolution studies. It excels at covering large areas quickly, optimizing field data acquisition resources while providing accurate and detailed environmental representation to support various analysis and planning tasks.

On the other hand, for the documentation of the underground elements, the use of a Wearable Mobile Mapping System (WMMS) based on LiDAR technology was implemented. This system enables the capture of three-dimensional data in motion using high-precision LiDAR sensors, combined with inertial units (IMUs) and GNSS positioning systems or relative positioning techniques. Its application is especially advantageous in difficult-to-access environments, where the installation of static equipment is not feasible or efficient [53]. Thanks to this methodology, it is possible to generate detailed 3D models in reduced time, optimizing data acquisition and improving the geometric documentation of the subway environment with high spatial accuracy.

The main criterion in the choice of these techniques has been the optimization of time and resources, ensuring a balance between the accuracy of the data obtained and the operational feasibility of the project.

- **Aerial Photogrammetry**

For the documentation of the LTCP area, traditional aerial photogrammetry using a drone was employed, a technique widely used in the field of geomatics due to its ability to generate detailed 3D models and highly accurate derived products. For this work, the Mavic 2 Pro drone (DJI company, Nové Město, Czech Republic) was used, a compact and highly efficient device equipped with a 20-megapixel Hasselblad L1D-20c camera and a 1-inch CMOS sensor.

The flight mission was pre-planned using PIX4Dcapture Pro APK v.4.11.0 [54], ensuring comprehensive coverage of the region of interest (ROI) and optimizing the quality of the acquired data. During planning, several determining factors for efficient image capture were considered, including the autonomy of the drone, its sensor quality and its stability in adverse conditions. In addition, the extension and location of the study area were taken into account, ensuring homogeneous data acquisition and complete coverage to optimize the performance of subsequent processing. A key aspect in this phase was the estimation of the flight time and the number of batteries required, which enabled efficient management of resources to ensure the completion of the mission without interruptions. Technical parameters were also defined to optimize image capture, establishing a 70% overlap in the direction of flight and 40% in the lateral passes, configuring the flight in a grid pattern. This strategy ensured that each surface point appeared in at least four images, an essential condition to achieve an accurate three-dimensional reconstruction of the terrain and guarantee high-quality photogrammetric results.

Another relevant factor that was taken into account was the weather, paying special attention to the prevailing winds, since the LTCP is located in an area close to the sea.

On the other hand, prior to the flight, control targets were distributed uniformly in the region of interest (ROI), selecting strategic locations that allowed their clear identification in the images captured during data acquisition with the drone. These points served as Ground Control Points (GCPs), key elements for the external orientation and accurate georeferencing of the generated model. To ensure maximum accuracy in the results, the GCPs were captured by a complementary topographic survey using a high-precision Global Navigation Satellite System (GNSS). In this case, Topcon GR-5 was used, which offers an accuracy of ±1 cm. Incorporating these points reduced geometric distortions in the reconstruction process and significantly enhanced the absolute accuracy of the resulting photogrammetric model.

In addition, at the locations where the sensors were installed, aiming targets were placed to ensure their correct spatial location within the photogrammetric model.

Once the images and GNSS data were acquired, the information was processed using specialized photogrammetric software, applying block adjustment algorithms and aerial triangulation to generate accurate three-dimensional models. This approach yielded a Digital Surface Model (DSM) and derived products, such as high-resolution orthophotographs, which will be used for further analysis.

**Figure 9:**
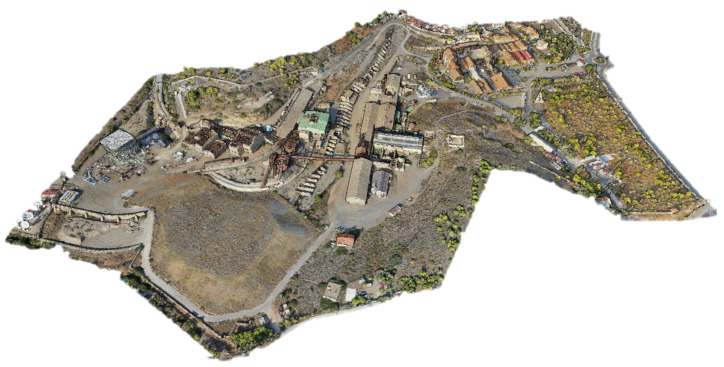
Three-dimensional point cloud generated with the mentioned photogrammetric techniques.

During the mission, 1899 images were captured from a flight altitude of 60 m, in an approximate time of 40 min, covering an area of approximately 24 hectares and achieving a Ground Sample Distance (GSD) of 1.53 cm. These images were processed using the photogrammetric reconstruction software program GRAPHOS v.2.0.0.beta.8 (inteGRAted PHOtogrammetric Suite) [55], which enables the generation of high-quality 2D and 3D metric products, such as orthophotos, Digital Elevation Models (DEMs), and a dense point cloud (Figure 9).

Together, the combination of aerial photogrammetry and high-precision GNSS georeferencing ensured that a detailed and reliable model of the LTCP could be obtained, optimizing the geometric documentation of the site with an efficient and accurate approach.

UAV-based photogrammetry was chosen over traditional topographic surveys or satellite imagery for its ability to capture high-resolution (sub-centimeter) orthophotos and dense point clouds at relatively low cost and with high temporal flexibility. The approach allows repeated acquisition at short intervals, enabling change detection of surface features. Compared with satellite imagery, UAV photogrammetry offers higher spatial resolution and full control over acquisition parameters, which is critical to detecting small-scale deformations in the landfill cover.

- **Wearable Mobile Mapping System**

For the documentation of underground areas (where limited access and poor visibility can hinder data acquisition), a Leica BLK2GO handheld laser scanner was used. This device integrates LiDAR technology along with visible imaging cameras, enabling real-time three-dimensional capture as the operator moves through the environment. Its portable design and autonomy make it an ideal tool for documenting tunnels, galleries, and other subway spaces where traditional techniques may be inefficient.

The data acquisition process with the Leica BLK2GO was carried out following a structured methodology to ensure maximum accuracy and efficiency in the documentation of subway spaces. Initially, a pre-planning phase was conducted to define scanning paths based on the spatial layout, aiming to guarantee complete coverage and minimize data voids. Subsequently, the scanner was configured, adjusting the capture parameters to optimize point density and acquisition speed, achieving a balance between quality and scanning time.

During data acquisition, the operator traversed the areas of interest while carrying the BLK2GO, which captured information in real time without requiring fixed reference points. Leveraging its SLAM (Simultaneous Localization and Mapping) trajectory sensor, the scanner continuously reconstructed the environment in three dimensions, dynamically adapting to changes in spatial geometry. The system integrates three 4.8-megapixel global shutter cameras for real-time visual navigation, along with a 12-megapixel rolling shutter camera for high-resolution image capture, enriching the point cloud with color and additional contextual detail.

Once the scanning was completed, data were reviewed and processed in Leica Cyclone Register 360 Plus (BLK Edition), where noise filtering, alignment, and georeferencing were performed to ensure data quality and enable integration with other geometric models.

The Leica BLK2GO operates with an 830 nm wavelength laser, with a field of view of 360° on the horizontal axis and 270° on the vertical axis, enabling enveloping data capture. Its measurement range is between 0.5 m and 25 m, with an acquisition rate of 420,000 points per second and a noise level of ±3 mm. In addition, the device features the BLK2GO Live application, which enables real-time visualization of the scan trajectory, capture progress, and 360° images obtained during the process. In terms of design, the BLK2GO features a light weight of 775 g (1.7 lbs), making it easy to handle in complex environments, and a battery with a battery life of up to 45 min of scanning per charge, ensuring efficient acquisition without the need for constant replacement.

**Figure 10:**
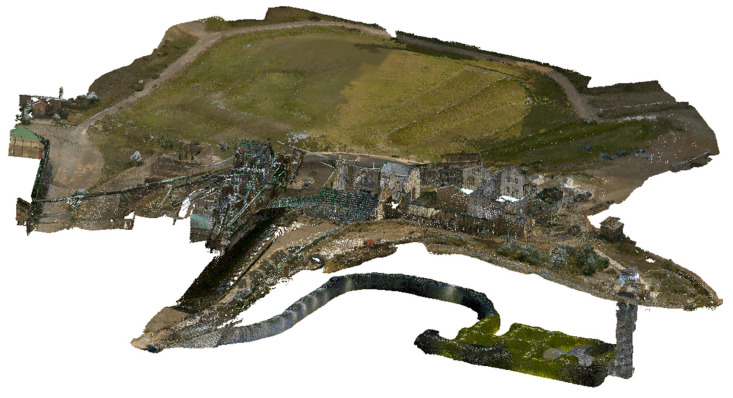
Point cloud generated with WMNS.

Data capture with the BLK2GO scanner took approximately 25 min, enabling complete geometric characterization of the underground gallery. Additionally, exterior zones shared with the photogrammetric survey were included to create overlapping areas, facilitating the integration and fusion of both datasets. The final product is presented in Figure 10.

The BLK2GO Wearable Mobile Mapping System was selected because it enables fast, operator-friendly acquisition of 3D point clouds inside narrow or complex structures where tripod-based terrestrial laser scanners are impractical. Its SLAM technology ensures continuous georeferencing without requiring targets or control points along the trajectory. Compared with conventional TLS, this WMMS reduces fieldwork time by up to 70% and facilitates frequent re-scans for monitoring structural changes.

- **Merging of point clouds**

Once both point clouds obtained through aerial photogrammetry and handheld laser scanning were processed, the next step involved merging them to generate a seamless and coherent 3D model of both the surface and underground environments.

The point cloud generated from aerial photogrammetry was used as the reference base for the alignment process, as it was georeferenced in absolute coordinates by using high-precision GNSS-surveyed GCPs. In contrast, the data captured by the Leica BLK2GO handheld scanner were in a relative local coordinate system, specific to the device, requiring a registration process for integration.

For the merging, CloudCompare v. 2.13.2 software was used, applying the ICP (Iterative Closest Point) algorithm [56]. An initial alignment was performed using manual point pair picking, identifying common features in overlapping areas, primarily in outdoor zones covered by both the photogrammetric and laser scanning campaigns. This initial step provided a rough spatial approximation between the datasets.

Subsequently, the automatic ICP algorithm was applied, which iteratively refined the position of the local (BLK2GO) cloud to minimize the mean distance to the nearest points in the reference (photogrammetric) cloud. This process yielded high-precision alignment with a residual error of less than 2 cm, which is considered acceptable for the intended applications.

Finally, the merged point cloud was trimmed: the surface area was preserved using the photogrammetric point cloud, while the interior sections were retained from the WMMS dataset. This selective refinement ensured the highest geometric quality for each part of the model, optimizing both spatial accuracy and visual coherence. Subsequently, the two-point clouds were combined using the “Merge” tool in CloudCompare, resulting in a single, unified dataset that seamlessly integrates surface and subsurface elements. The option to retain a scalar field indicating the origin of each point was intentionally omitted, as the objective was to produce a fully cohesive and integrated model.

**Figure 11:**
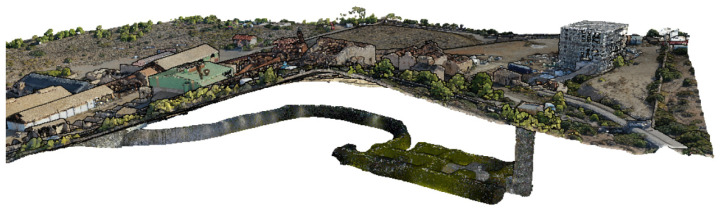
Merged 3D point cloud integrating surface data from aerial photogrammetry and subsurface data from the WMMS.

This methodology resulted in a high-density, georeferenced point cloud suitable for further modeling, structural analysis, and visualization within the interactive Digital Twin platform (Figure 11).

#### 2.2.3. Interior Model Using Geophysical Techniques

Among the various geophysical techniques that can be applied in studies of this nature, Ground-Penetrating Radar (GPR) and magnetometry were selected. The selection of these methodologies responds to the specific characteristics of the study area, in particular the presence of an artificial landfill. In this context, Electrical Resistivity Tomography (ERT) was deemed unsuitable due to the heterogeneity of the materials and the irregular distribution of waste, which would have hindered the acquisition of reliable data. Consequently, alternative techniques better suited for detecting subsurface anomalies and characterizing existing structures were selected.

- **Ground-Penetrating Radar prospecting**

The GPR survey was focused on the characterization of the surface layers of the deposit, with special attention to the detection of the first geotextile layer and the identification of possible internal anomalies associated with water accumulations or structural failures. For this purpose, a Proceq GS8000 high-resolution georadar system was used, equipped with an Ultra-Wideband (UWB) antenna operating in a frequency range of 40 to 3440 MHz.

Data collection was carried out on the surface, following a grid of semi-structured transects, adapted to the topographic and environmental conditions of the landfill, including sparse vegetation and irregular surfaces. The GPR system was moved manually, synchronized with GNSS sensors for accurate georeferencing. A complete sweep of the repository was performed with special emphasis on the perimeter areas, where a greater vulnerability of the isolation system was suspected.

The georadar data were processed using GPR Insights software by Screening Eagle, following a workflow designed to maximize the quality and usefulness of the geophysical information. First, topographic correction and precise synchronization with the GNSS data were applied to ensure the proper georeferencing of the profiles. Next, gain filtering and background noise removal were performed to improve the signal-to-noise ratio. Subsequently, the Hilbert transform was applied, which made it possible to highlight more clearly the horizontal reflections associated with structural interfaces such as the geotextile layer. The profiles were then three-dimensionally interpolated to generate a volumetric (3D) model of the subsurface, which facilitated continuous spatial analysis. Finally, a detailed signal analysis was carried out, focusing on the estimation of the depth of the reflecting interfaces and the depth of the reflecting interfaces [57].

Analysis of the GPR data revealed a heterogeneous distribution of the first geotextile layer, with variable depths ranging from 40 to 150 cm and a shallow outcrop observed on the eastern perimeter, which could compromise the effectiveness of the isolation barrier. In addition, low-attenuation electromagnetic anomalies associated with the presence of moisture or seepage were detected, mainly in the marginal areas of the reservoir, suggesting possible deficiencies in the drainage system or the aging of the geotechnical material [47].

GPR was selected over alternative geophysical methods such as ERT because it provides higher-resolution profiles and faster coverage of the entire site, which is essential to detecting shallow anomalies such as geotextile layer failures. GPR is non-invasive, does not require electrode installation, and delivers immediate visual feedback in the field, making it highly suitable for integration with other real-time monitoring data within the Digital Twin.

- **Aerial magnetometry with drone**

The magnetic survey was carried out using an airborne system based on a multirotor drone, which was coupled with a high-sensitivity vector magnetometer. For this task, a GEM Systems GSMP-35U magnetometer was used, a piece of equipment based on high-resolution proton precession sensors capable of recording magnetic field variations with a sensitivity of up to 0.01 nT and a sampling frequency of up to 10 Hz, which allows for the accurate detection of small magnetic anomalies generated by ferrous materials or alterations in the internal structure of the deposit.

The magnetometer was mounted on a rigid non-magnetic suspension several meters below the drone to minimize electromagnetic interference generated by the UAV motors and electronics. The UAV system used was a six-rotor drone with a payload capacity of more than 3 kg and sufficient autonomy for flights of up to 20 min per battery, which made it possible to cover large areas in each session.

Flight planning was performed using autonomous navigation software, defining a grid of parallel lines (transects) regularly spaced between 1.5 and 2 m, at a constant altitude of 2.5 m above the ground, following the recommendations for high-resolution surveys. To ensure topographic accuracy and data georeferencing, the drone was equipped with a GNSS RTK receiver and an inertial system (IMU).

The collected magnetic data were subsequently corrected for diurnal effects by comparison with a fixed base station and treated with pole reduction filters, gradient analysis, and the inverse 3D modeling of the magnetic susceptibility. The final result was a 3D model that reflects, with high resolution, the internal magnetic anomalies of the deposit, enabling the identification of areas with accumulation of ferromagnetic metals (such as iron or manganese) and zones with low magnetic response associated with diamagnetic compounds such as lead, arsenic, or zinc [46]. This UAV–magnetometric approach offers an efficient, safe, and non-invasive alternative to traditional ground-based methods, especially useful in environments with irregular topography, presence of vegetation, or limited access.

UAV-based magnetometry was preferred over ground magnetometer surveys to enable uniform coverage of the landfill surface with minimal human exposure to potentially hazardous zones. Airborne data collection improves efficiency, especially for large areas or rough terrain, and produces consistent data grids that can be easily co-registered with photogrammetric and GPR results. This method is particularly effective for detecting buried metallic objects or anomalies indicative of waste material heterogeneity.

### 2.3. Platform Development

As outlined in previous sections, the aim of this research study is to develop an experimental interactive Digital Twin using advanced virtual reality technologies. This digital model enables exploration, analysis, and management of a restoration environment by integrating multiple data sources into a dynamic and interactive virtual platform.

At the core of this technology is a descriptive data model, structured in a MariaDB database, which organizes and manages all the information collected. The platform performs real-time queries to this database, applying predefined rules to extract and dynamically represent critical environmental parameters, enabling the continuous monitoring of the environment and the visualization of variations in the conditions detected.

For its development, multiple sources of information (such as the previously identified ones) are integrated into a unified virtual environment. This environment is structured around panoramic images, which serve as the primary interface, with the various datasets linked to specific locations within them. The technical implementation was carried out using Pano2VR^®^ (https://ggnome.com/pano2vr/, accessed on 1 June, 2025)), a cost-effective tool that enables the creation of interactive virtual tours.

The development of the platform required the programming of a customized web interface using standard languages, such as HTML for content structuring, CSS for visual design, and JavaScript to provide interactivity and dynamic functions to the system. The GUI was structured in two main sections:

- A header, intended to display information about the entities participating in the project.
- A main body, where interactive panoramas are projected, spatially linked data are integrated, and a site map with navigation options is displayed.

To improve user interaction, HTML-based hotspots with JavaScript functionality were added, providing contextual information through images, data, or external links. The JavaScript navigation menu offers features such as panorama rotation, zoom, site map viewing, and access to PDF documentation via iframes.

One of the most innovative elements of the Digital Twin is the integration of 3D point clouds rendered in real time, using the open-source Potree^®^ library, based on WebGL. This technology enables efficient visualization of large volumes of geometric data, optimizing performance without compromising quality. In addition, Potree^®^ offers advanced metric tools, such as distance measurement, and area and volume calculation, key functionalities for professionals involved in conservation and technical site analysis.

The connection between the 3D rendering engine and the virtual environment was solved by integrating an HTML page embedded in the platform. It loads the Potree viewer, which allows users to interact with the point cloud in real time, obtain coordinates, perform geometric measurements, or analyze the spatial distribution of the environment.

This comprehensive approach, which combines virtual reality technologies, three-dimensional modeling, and real-time data integration, enabled us to create an interactive Digital Twin capable of offering an innovative tool for the exploration, interpretation, and proactive management of the site under restoration.

#### 2.3.1. Panoramic Images

As noted above, the primary interface is based on panoramic images. After a detailed analysis of the different options for capturing these images, an SLR camera equipped with a fisheye lens was selected. This technique is based on taking seven photographs from the same position: six at 60° intervals and one overhead shot. This option was selected due to the advantages offered by DSLR equipment over 360° cameras, which automatically generate panoramas. Specifically, DSLR systems enable precise control of imaging parameters such as ISO, exposure, and white balance while also ensuring superior image quality.

Image acquisition was carried out using a Nikon D5600 camera (Nikon, Shanghai, China) equipped with a Nikkor 10.5 mm f/2.8G lens, capable of capturing images at a resolution of up to 24 megapixels. The camera was mounted on a photographic tripod with a Manfrotto 303SPH panoramic head, ensuring precise capture from predefined angles. Prior to the image acquisition process, calibration of the camera–lens system was performed on the panoramic head, aligning the nodal point with the axis of rotation to eliminate potential parallax errors. In addition, internal parameters such as aperture, exposure time, and ISO sensitivity were configured, and the activation of HDR mode was evaluated as part of the setup.

**Figure 12:**
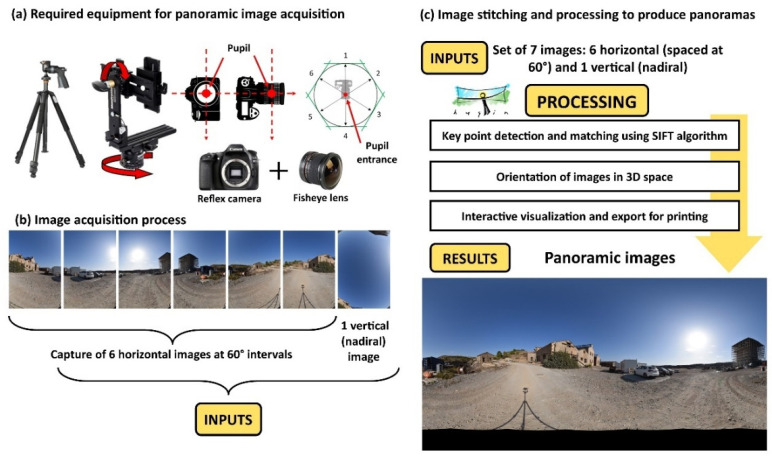
Workflow for panoramic image generation, from acquisition and orientation to visualization and generation.

Once the images were obtained following the mentioned protocol, they were processed with the open-source software program Hugin [58]. The workflow (presented in Figure 12) started with the extraction and matching of key points between individual images using the Scale-Invariant Feature Transform (SIFT) algorithm. Subsequently, feature matching was performed using the L2 norm and the Random Sample Consensus (RANSAC) algorithm, following the approach by Brown and Lowe [59], in order to calculate the homography matrix between individual shots. Then, a global registration phase was carried out to minimize the accumulation of errors, optimizing the camera distortion parameters, focal length, and angular positions of each image. Finally, the images were projected onto a spherical coordinate system to generate the final panorama. During this process, exposure compensation and multiband fusion techniques were applied to improve the radiometric coherence of the resulting image [60].

Additionally, three aerial panoramas were acquired using a Mavic 2 Pro drone from different vantage points, providing a more comprehensive and detailed view of the LTCP as a whole. These panoramas were automatically generated by the drone’s onboard software, eliminating the need for further processing.

In total, 27 ground-level images and 3 high-resolution aerial panoramas in .tif format were captured from key locations within the LTCP restoration area. The documentation encompassed (i) the landfill under study, with particular attention to the precise positioning of the sensors; (ii) the various structures located within the restoration zone; (iii) the underground gallery; and (iv) a series of aerial views offering an overarching perspective of the LTCP, supporting detailed spatial analysis. This integrated approach enables a more accurate and comprehensive representation of the current condition of the complex.

#### 2.3.2. Descriptive Data Model

A descriptive data model was developed to structure and integrate heterogeneous information relevant to the LTCP study area. This model consolidates geophysical, environmental, structural, documentary, and historical data from diverse sources (including IoT sensors, GPR, magnetometry, and expert inputs), to enable a detailed and coherent representation of the restoration environment. Data correlation within the model is achieved through a modular relational structure, inspired by semantic frameworks typically used in spatial decision support systems.

We considered three possible approaches for the construction of the data model: (i) temporal and spatial integration of heterogeneous data, (ii) semantic linkage through common attributes, and (iii) modular structure for cross-analysis and interpretation [61,62]; this research study adopted the first strategy as the primary integration method. This choice was based on the characteristics of the available datasets, which are primarily georeferenced or spatially localized (e.g., sensor locations, GPR profiles, magnetometric measurements, and photogrammetric point clouds). Given this spatial nature, integrating data within a common two-dimensional grid proved to be the most practical and effective approach to aligning and correlating inputs from multiple acquisition technologies.

The selected method enabled the implementation of a dynamic voxelization framework, where the study area was divided into square spatial units (voxels). Each voxel functions as a spatial container that stores attributes from different data sources, either directly measured (e.g., elevation or geophysical readings) or interpolated (e.g., environmental sensor values). This voxel-based structure facilitates consistent data alignment across technologies, using spatial and, where applicable, temporal references [63].

Compared with semantic or modular approaches (which require deep ontological definitions or the partitioning of information into separately managed subsystems), spatial integration offers a more direct, scalable, and operationally efficient method for correlating multi-source data. It enables localized analysis at the voxel level while preserving flexibility for future model expansion.

Ultimately, this strategy provides a robust foundation for advanced spatial analysis, continuous environmental monitoring, and the development of a functional and interactive Digital Twin of the restoration site.

- Temporal and spatial integration of heterogeneous data

To enable effective correlation among heterogeneous data sources, a 2D voxelization strategy (more precisely, a 2.5D grid-based approach) was implemented. The study area was divided into a regular grid of square units (voxels) of 5 × 5 m, each functioning as a planar spatial unit for the integration and organization of the collected information. While the grid is structured primarily in two dimensions (X and Y), each voxel is also associated with elevation-related attributes (Z) derived from 3D data sources, such as photogrammetric point clouds, enabling a pseudo-volumetric characterization of the terrain and subsurface.

Each voxel serves as a data container, storing attributes from multiple sources (environmental sensors, geophysical surveys, and remote sensing techniques) standardized using spatial and, when applicable, temporal criteria. This standardization ensures coherence in data assignment and supports integrative analysis.

Rather than using a formal geodetic coordinate system, spatial referencing is achieved through discretization: the dataset is divided into fixed spatial cells based on their geographic location within the grid. This approach enables the co-location of data acquired through different technologies, even when these vary in spatial resolution, coordinate systems, or acquisition geometry. As a result, each voxel represents a common analysis unit where multiple observations can be jointly evaluated, facilitating efficient cross-referencing and multi-source integration.

The centroid of each voxel serves as the reference point for assigning or interpolating data values. The following attributes are stored or estimated within each voxel:

- **Surface elevation**: Computed as the mean height of the photogrammetric point cloud contained within the voxel.
- **Water accumulation potential**: Inferred from GPR signal anomalies, particularly attenuation zones, low-amplitude reflections, or hyperbolic patterns indicative of subsurface moisture. These are spatially interpreted to estimate the likelihood of water presence.
- **Presence of geotextile layers**: Detected through characteristic GPR reflection signatures. Two attributes are stored per voxel, one for surface or near-surface layers and another for deeper layers, based on signal amplitude and continuity within defined depth intervals.
- **Metallic mineral content**: Estimated from magnetometric measurements, through the analysis of magnetic susceptibility or localized magnetic anomalies. Average intensity values are assigned to the voxel, aiding in the identification of ferrous or conductive materials potentially linked to contamination or structural features.
- **Environmental parameters**: Variables such as nitrogen (N), phosphorus (P), potassium (K), soil moisture, soil temperature, electrical conductivity (EC), and salinity are measured through a network of IoT sensors deployed across the study area. As these sensors do not provide full spatial coverage of every voxel, the values for each voxel centroid are estimated using an Inverse Distance Weighting (IDW) interpolation method [64]. The interpolated value V_C for a given voxel C is computed as (Equation (1))

(1) $$V_{C}=\frac{\sum_{k=1}^{n}\left(\frac{1}{{\left\|C-S_{k}\right\|}^{p}}\cdot v_{k}\right)}{\sum_{k=1}^{n}\frac{1}{{\left\|C-S_{k}\right\|}^{p}}}$$

where $v_{k}$ is the parameter value measured by sensor k; $\left\|C-S_{k}\right\|$ is the Euclidean distance between the voxel centroid and sensor k; and p is the power factor controlling the distance influence (commonly set to 2).

**Figure 13:**
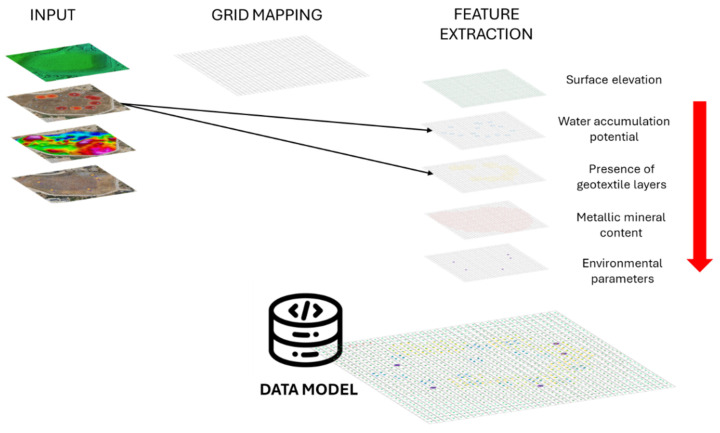
Voxel-based integration of multi-source spatial data.

**Figure 14:**
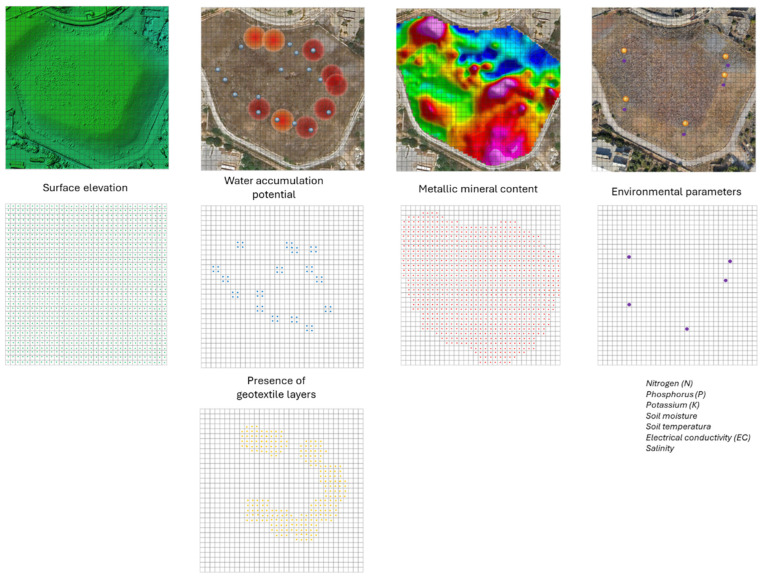
Voxel-level attribute assignment by variable type.

Below, Figure 13 and Figure 14 describe the data and their structuring within the model.

This method ensures that measurements taken closer to a given voxel have greater influence on its estimated value, thus enabling a spatially continuous and locally relevant representation of environmental conditions throughout the grid.

Overall, this voxel-based approach enables the creation of a functional, adaptable, and scalable correlation system. Rather than merely overlaying data, the model integrates observations by using logical interpretation rules, supported by expert knowledge and empirical patterns. As a result, it not only offers a detailed and reliable description of the current state of the restoration environment but also facilitates the early detection of anomalies, potential contamination risks, and the prediction of future system behavior, endowing the Digital Twin with actionable value well beyond static visualization.

- **Data Storage and Monitoring Logic**

The data generated from each voxel (including raw values and interpolated parameters) is systematically stored in a spatially referenced relational database. Each record in the database represents a unique voxel and includes X/Y coordinates, elevation, and all associated attributes, ranging from GPR-based moisture indicators and magnetometric readings to interpolated environmental variables from IoT sensors.

This structure enables efficient spatial querying, time-series analysis, and linkage to the Digital Twin platform for visualization and simulation.

To operationalize these data for conservation purposes, the system incorporates a logic for automatic status classification using Key Performance Indicators (KPIs), following strategies similar to those developed by Mora et al. [65] in HBIM environments.

Each monitored or interpolated variable is compared against material-dependent threshold values, and the percentage of measurements within acceptable limits is calculated as (Equation (2))

(2) $$KPI=\frac{N_{in}}{N_{total}}$$

where $N_{in}$ = number of measurements within defined tolerances and $N_{total}$ = total number of measurements in the analysis period.

This mechanism enables automatic alerts in the Digital Twin interface. For instance, if a voxel shows low KPI scores for moisture or salinity near geotextile layers, it may trigger a warning about possible infiltration or material degradation.

Additionally, different thresholds and KPIs can be customized per material type (e.g., for areas with organic materials or metallic inclusions) following PAS 198:2012 [66] standards or empirical calibration based on historical observations.

- **Voxel Data Storage Architecture and Integration into the Digital Twin System**

These voxel-level data are systematically stored in a relational geospatial database hosted on a secure MariaDB server, with each voxel being represented as a unique record linked to spatial coordinates (X, Y) and associated attributes (e.g., elevation, geophysical signatures, and interpolated environmental parameters). The structure follows a modular schema, where each data source (GPR, magnetometry, UAV-derived photogrammetry, and IoT sensors) is managed in a dedicated table and connected through foreign keys referencing the voxel grid.

The database is continuously updated via scheduled synchronization scripts and API connections to field-deployed IoT devices. Data integrity is ensured through automated validation routines and timestamped entries, enabling not only the spatial but also temporal tracking of changes. This design supports temporal querying and longitudinal analysis, which are crucial to monitoring the evolution of environmental conditions over time.

Furthermore, the database acts as the backend engine of the Digital Twin, feeding the visualization platform and alert system with real-time and historical data. Analytical modules can retrieve information at the voxel level to perform KPI assessments, risk detection, or predictive modeling tasks.

This centralized, scalable, and queryable database architecture ensures high-performance access, cross-source interoperability, and the long-term sustainability of the Digital Twin system.

In a final step, a KPI-based alert system was established to notify the user of potential anomalies in the monitored tank. These anomalies mainly concern abnormal variations in water content (either increases or decreases), as well as possible fluid leaks, migrations, or concentrations of specific chemical elements that fall outside the defined normal ranges.

### 2.4. Additional Data and Site Context

One of the essential aspects of coherent platform development is to obtain a deep and detailed understanding of the site under study. Achieving this requires a rigorous and well-structured data collection process, which is often challenging due to the dispersed, diverse, and heterogeneous nature of the available information. Relevant information often comes from diverse and fragmented sources, making it difficult to locate it, access it, and retrieve it comprehensively. Data may vary in format and be dispersed across different repositories, requiring standardization for effective integration. To address these challenges, it is key to define clear search criteria and specific objectives before data collection. Once gathered, the information must be homogenized and structured to enable coherent analysis, comparison, and integration into databases or modeling systems.

In this research study, the collection of information was structured in two specific main approaches. On the one hand, efforts focused on complementing existing information to develop a comprehensive descriptive data model. For this purpose, data collection was structured around three key areas: (i) Historical mining activities in the region, for which the process was relatively straightforward due to the extensive prior knowledge available—over the years, different studies and projects have produced a well-documented and robust database, enabling a reliable reconstruction of the area’s mining evolution. (ii) Restoration works carried out in recent years, thoroughly documented through detailed technical reports that describe each phase of the reclamation process, thus ensuring access to accurate and well-structured information. (iii) Environmental monitoring and pollution control, supported by the LTCP’s consolidated database of periodic environmental measurements—these records are essential, especially in relation to the evaluation of geophysical results and their integration into system environmental analysis.

On the other hand, a process of debugging and selection of the information collected was carried out with the aim of integrating only the most relevant data in the digital platform. In this process, the exclusion of sensitive or confidential data was guaranteed, prioritizing those that provide value for the understanding and visualization of the state of the area under restoration. In addition, in order to complement the textual information and facilitate the understanding of the context, extensive visual documentation was performed through the capture of multiple images in different formats and from different perspectives. These images not only enrich the project documentation but are also essential to their integration into interactive tools for exploration, analysis, and result dissemination of the study area.

### 2.5. Accuracy Assessment and Validation

To guarantee the reliability of the results, the accuracy of the different data acquisition and processing stages was systematically evaluated. The photogrammetric model was georeferenced using ten GNSS-surveyed GCPs obtained with a Topcon GR-5 receiver in RTK mode. The resulting point cloud achieved an absolute horizontal accuracy of ±1.5 cm and a vertical accuracy of ±2.0 cm, which were validated against an independent set of five checkpoints not used for model adjustment.

The BLK2GO wearable LiDAR point clouds were subsequently aligned using ICP algorithms in CloudCompare. The mean residual alignment error obtained was below ±2 cm, fully consistent with the manufacturer’s specifications and ensuring reliable 3D reconstruction of the interior spaces.

In order to validate the interpretation of the GPR data, three test pits were excavated along the eastern perimeter of the landfill, coinciding with anomalies that suggested the existence of partial damage to the first geotextile layer. The measured depths of the layer matched the GPR-derived depths with a mean absolute error of 6 cm, confirming the reliability of the radargram interpretation and supporting the conclusions drawn from the geophysical survey.

Finally, all NPK-7in1 sensors were calibrated prior to deployment using certified soil reference solutions. The calibration results were cross-checked with portable field instruments, yielding deviations below 5% for moisture and salinity measurements and below 3% for NPK concentrations. This systematic validation provides confidence that the geometric, geophysical, and sensor-derived data integrated into the Digital Twin are robust and suitable for long-term monitoring and decision support.

## 3. Experimental Results

As previously indicated, the selected case study focuses on a specific area within the Lavrion Technological and Cultural Park (LTCP), which is currently undergoing restoration. The analysis specifically targets a permanently sealed hazardous-waste landfill, whose environmental management demands continuous and precise monitoring due to the potential environmental risks it poses.

### 3.1. Fieldwork

The results of the data collection campaigns conducted for the case study are presented in this section, based on the methodology outlined in the preceding subsections.

#### 3.1.1. Monitoring System

As discussed before, five NPK-7in1 sensors, together with a Dragino DLOS8N Gateway, were installed to assess the landfill conditions, enabling the continuous and accurate monitoring of soil quality using long-range, low-power communication technology. In this way, continuous field-recorded information is available at several of the most strategic and complex locations within the evaluated repository.

In addition, as part of the monitoring system, an interactive web platform has been developed, accessible via a public link (https://georoad.usal.es/lavrio/ access on 16 September 2025). This tool enables real-time visualization of data collected by the sensors, offering users an efficient way to analyze the information, both individually and comparatively. It also allows data to be downloaded in CSV format for further analysis using specialized tools.

**Figure 15:**
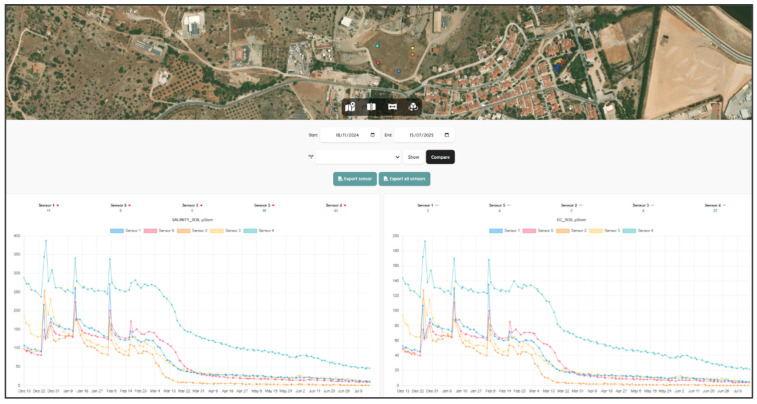
Main window of the IoT monitoring platform.

As can be observed below in Figure 15, the platform also incorporates advanced tools like dynamic and interactive visualizations that support the analysis of soil parameter trends and the detection of anomalies. Additionally, it features a map displaying the geolocation of each sensor, offering spatial context to enhance the interpretation of the collected data.

The developed system includes a responsive frontend built with HTML, CSS, and JavaScript, optimized for field use on different devices. The backend, based on an Apache server with PHP and MariaDB, handles data processing and storage, with JSON enabling efficient communication between components. Fully customizable and standalone, the platform supports integration with tools like WebGIS or virtual tours. It also includes a dark mode option to enhance user comfort.

**Figure 16:**
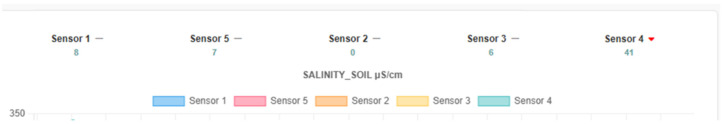
Soil salinity readings (SALINITY_SOIL, µS/cm) recorded by five IoT sensors distributed in the study area. The image shows a comparative panel with the soil salinity values detected by five different sensors (Sensor 1 to Sensor 5). Each sensor is represented by a different coloured bar, with the corresponding salinity value indicated above each label.

As seen in Figure 16, the soil salinity values recorded by the sensors vary significantly across the monitored locations. Most sensors (Sensors 1, 2, 3, and 5) report low salinity levels, ranging from 0 to 8 µS/cm, well below critical thresholds typically associated with soil degradation or plant stress. In contrast, Sensor 4 stands out with a markedly elevated value of 41 µS/cm, which exceeds the commonly accepted limit for optimal soil conditions (usually <30 µS/cm in many agricultural or environmental restoration contexts).

This abnormal reading, highlighted by a red indicator on the platform, triggers a warning event in the Digital Twin system, as defined by the KPI logic established for environmental risk detection. When the threshold is surpassed, the voxel or sensor node is automatically classified as high-risk, enabling proactive diagnostics and targeted field intervention.

This intelligent alerting system based on KPI thresholds provides critical support for decision-making processes. Combined with the spatial visualization tools integrated in the platform (e.g., sensor maps, time-series trends, and data overlays), it enables technical teams to monitor site conditions dynamically, detect early signs of degradation, and act promptly to prevent further environmental deterioration.

#### 3.1.2. Digitalization

As a result of the geometric digitization process of the Park, high-precision three-dimensional cartographic products that enable the visualization, analysis, and interpretation of the state of the environment, both on the surface and underground, have been generated. These products have been integrated into interactive online platforms that facilitate their exploration and use by technicians, researchers, and environmental managers.

- **Point cloud data of both exterior and interior spaces**

The combination of aerial photogrammetry and handheld laser scanning generated a dense, georeferenced point cloud, representing in great detail both the surface environment of the reservoir and its underground gallery. This point cloud is accessible through the interactive Potree viewer, available at the following link: https://iedu.usal.es/lavrio/potree/index.html?ts=48530981 (access on 16 September 2025).

This viewer enables seamless 3D exploration, with built-in tools for taking metric measurements (distances, areas, and volumes), adjusting the level of detail displayed, and accessing sectional views or specific perspectives of the model. The two data sources (UAV and BLK2GO) were integrated through overlapping areas, ensuring seamless continuity between the exterior and interior 3D models.

**Figure 17:**
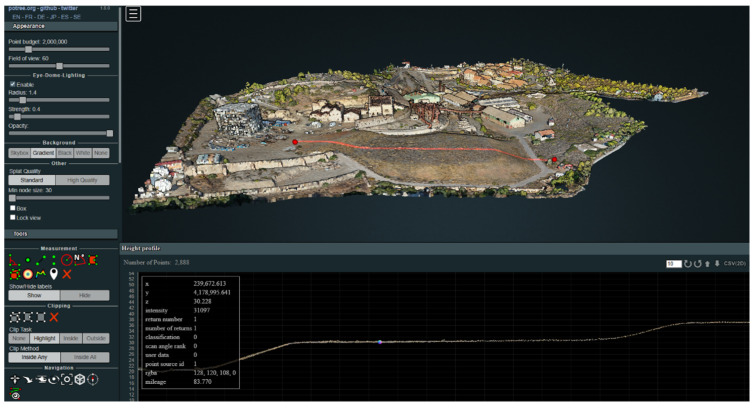
Visualization in Potree of the exterior and interior point cloud of the LTCP, with measurement and geometric analysis tools.

The point density and texture quality enable the identification of relevant elements such as surface irregularities, slopes, eroded edges, and interior structural details, making it a valuable resource for diagnostics and intervention planning. Below, Figure 17 presents the mentioned Potree viewer with the whole point cloud of the analyzed site.

- **Orthophotography and Digital Terrain Model**

Photogrammetric data were used to generate a high-resolution orthophotograph and a Digital Terrain Model (DTM), both of which were integrated into an interactive WebGIS platform developed with ArcGIS Experience Builder (https://experience.arcgis.com/experience/8a618cb2cc55485d83e52e568463a67b (access on 16 September 2025).

**Figure 18:**
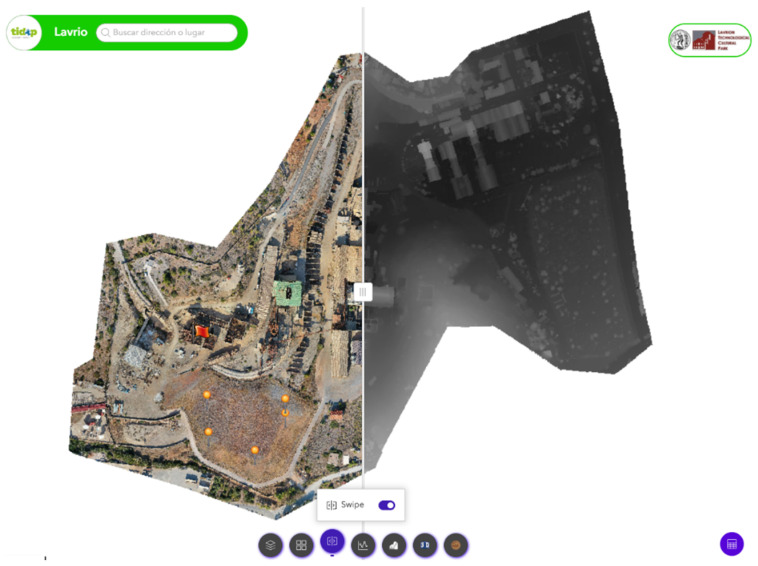
ArcGIS Web Experience interface with integrated orthophoto and DTM for interactive exploration of the LTCP.

This platform (shown in Figure 18) enables geospatial visualization of the study area, with tools for layer exploration, attribute query, view comparison, and interactive navigation. The orthophoto provides a detailed and up-to-date image of the reservoir’s surface condition, while the DTM enables the analysis of its morphology, slopes, accumulations, and potential runoff pathways. Both products have been georeferenced with centimeter precision, enabling their use in planning tasks, temporal comparison, and overlay with other sources of environmental or structural data.

#### 3.1.3. Subsurface Geophysical Investigation

**Figure 19:**
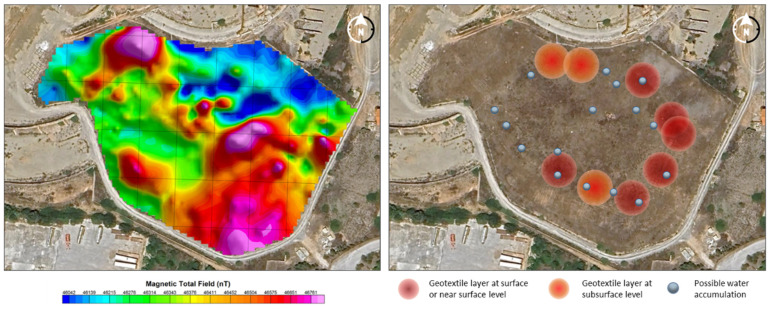
Results of the integrated geophysical analysis, showing the spatial distribution of mineral contents and the structural condition of the insulation layers.

The combined geophysical analysis has provided a comprehensive view of both the distribution of the deposit’s mineral contents and the condition of its insulation structures (Figure 19).

On the one hand, magnetometry has facilitated the development of a three-dimensional magnetic susceptibility model, identifying with great precision the areas of the deposit with the highest content of ferromagnetic materials (such as iron or manganese), as well as those areas dominated by diamagnetic materials (such as lead, arsenic, cadmium, or zinc), which present lower susceptibility. This information is crucial to assessing the distribution of contaminant residues and planning future selective or monitoring interventions [46].

On the other hand, GPR surveying has provided a detailed characterization of the condition of the first geotextile layer, one of the main components of the deposit’s insulation system. The analyzed layer presents a heterogeneous spatial distribution, with sectors where it is located at the expected depths and others (particularly along the eastern perimeter of the deposit), where it emerges at very shallow levels, potentially undermining its effectiveness as an insulating barrier. Furthermore, Ground-Penetrating Radar surveys have revealed electromagnetic anomalies compatible with the presence of water accumulation or infiltration, primarily in the peripheral zones of the reservoir. These findings may indicate deficiencies in the drainage system or progressive degradation of the insulating layers [47].

### 3.2. Virtual Platform-Based Digital Twin

**Figure 20:**
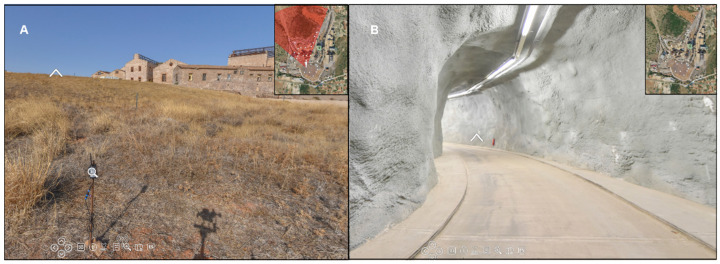
Captured views from the virtual tour of the LTCP infrastructure. (**A**) External deposit area showing the deployment of IoT sensor nodes. (**B**) Section of the underground gallery network.

As previously mentioned throughout the present manuscript, the final outcome of this research study is the development of a Digital Twin in the form of an interactive virtual tour of the mining case under study. This digital representation consolidates all the information gathered during the on-site data acquisition campaigns, including spatial data, sensor readings, and additional geophysical results. The resulting platform not only provides a comprehensive visualization of the LTCP infrastructure but also serves as a functional tool for analysis, monitoring, and informed decision making. In this sense, Figure 20 presents two different captured views of the mentioned virtual platform, which can be accessed through the following link: https://iedu.usal.es/lavrio/ (access on 16 September 2025).

**Figure 21:**
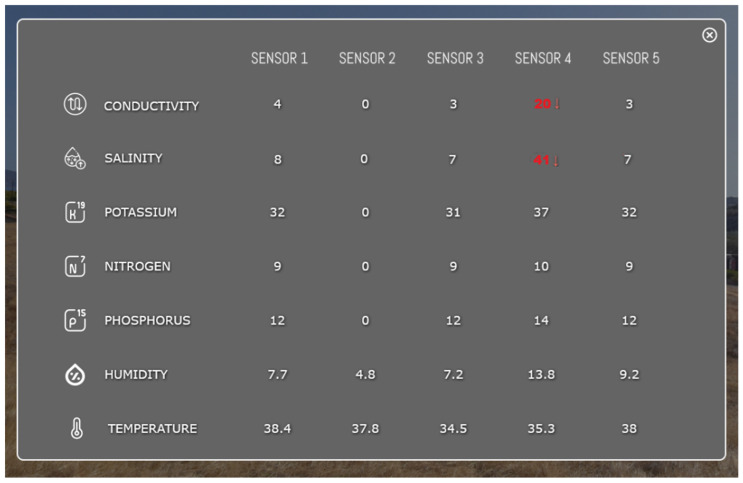
KPI-based alert system for IoT measurements included in the virtual platform.

All the main outputs described in the previous subsections were integrated into this platform:

- Three-dimensional photogrammetric and laser scanning models, displaying both the external morphology of the landfill and the interior underground galleries (point cloud visualizations, accessible through the embedded Potree viewer, enabling geometric inspection, measurements, and volumetric analysis).
- Interactive maps, integrating orthophoto, DTM, and environmental layers.
- Geophysical results, including magnetometric maps and GPR-based subsurface interpretations, which are embedded as spatial overlays or layers linked to specific hotspots.
- Real-time IoT sensor data, showing environmental variables such as soil salinity, temperature, or conductivity through dynamic charts and geolocated markers.
- KPI-based alert systems, configured to trigger warnings when specific thresholds are exceeded (e.g., high salinity and shallow geotextile exposure), providing an operational layer to the digital representation. An example of this alert system applied to IoT measurements is presented in Figure 21. As shown, the red values indicate anomalies or measurements that fall outside the predefined normal ranges established for the operating conditions of the analyzed tank.
- Information on the LTCP facilities to better understand their current status and past use.

By combining all these elements, the Digital Twin provides a spatially referenced, semantically enriched, and interactively accessible representation of the LTCP. The virtual tour is designed to be cross-platform and user-friendly, enabling stakeholders (including researchers, engineers, policymakers, and citizens) to explore the site, analyze relevant indicators, and interact with historical and real-time data in a fully immersive manner.

This platform is not a static 3D model but a living digital environment, continuously updated with data from the sensor network and open to the integration of new datasets, such as remote sensing imagery, remediation planning scenarios, or updated structural diagnostics.

Furthermore, the system architecture supports future expansion into immersive technologies such as VR, Augmented Reality (AR), and Mixed Reality (MR), enhancing its potential use in educational, participatory, and training contexts.

## 4. Discussion

In recent years, the application of Digital Twin technologies in post-industrial environments has gained significant traction, aligning with international sustainability agendas and the European Green Deal. This study contributes to that trend by demonstrating how digitalization, when integrated with environmental monitoring and heritage conservation, can unlock new opportunities for smart remediation, participatory governance, and cultural valorization. The integration of Digital Twin systems in heritage mining contexts fosters the emergence of “smart territories,” where environmental, cultural, and technological dimensions converge. These intelligent systems facilitate a real-time understanding of site dynamics and help align local restoration efforts with regional and European strategies focused on green transition, digital innovation, and rural revitalization.

The revaluation of mining environments as elements of industrial cultural heritage should extend beyond mere conservation efforts to include their reinterpretation and integration into contemporary societal frameworks. Acknowledging these sites as industrial heritage entails recognizing their historical, technological, and symbolic significance, thereby transforming them into dynamic spaces that embody the collective memory of human industrial development. Where conditions allow, mining areas can be repurposed into multifunctional facilities that serve current societal needs while simultaneously functioning as interpretive centers of historical knowledge. Through processes of rehabilitation, these spaces may evolve into open-air museums, cultural tourism destinations, or educational platforms that communicate the profound impact of mining on economic and technological advancement.

Such transformations not only preserve the tangible and intangible heritage associated with mining but also enable present and future generations to engage with and appreciate its contribution to the shaping of modern society, reinforcing a meaningful connection with this industrial legacy. Furthermore, the revaluation of historic mining sites can generate tangible benefits for local communities. Their rehabilitation from a cultural and tourism-oriented perspective offers significant economic potential through the promotion of industrial heritage tourism, environmental education, and scientific outreach. Beyond economic revitalization, such initiatives contribute to reinforcing the cultural identity of mining regions, enabling communities to reclaim and take pride in their historical legacy.

The accessibility of the digital platform developed in this study democratizes environmental information and fosters transparency. By enabling non-experts to visualize real-time sensor data, spatial patterns, and remediation progress, it supports environmental literacy and encourages communities to actively participate in the preservation and sustainable transformation of their surroundings. The methodological framework presented in this work serves as a transferable model for similar post-industrial or environmentally degraded sites. Its modular architecture (combining geospatial digitization, IoT monitoring, geophysical diagnostics, and interactive visualization) can be adapted to diverse geographical contexts, offering a scalable solution for integrated environmental assessment and heritage valorization.

In the mentioned context, this research study illustrates such potential through the case of the LTCP, where a former mining complex has been successfully repurposed into a hub for innovation, heritage interpretation, and public engagement. The LTCP exemplifies how industrial heritage, when integrated into a sustainable development framework, can become an active driver of social, economic, and cultural growth. Aligned with the ongoing efforts and initiatives of the Park, this work has developed a virtual environment of significant value for the future mining exploitation of the area and its outreach to the broader community.

The combination of low-power sensors with LoRaWAN technology facilitates continuous environmental monitoring, eliminating the need for frequent maintenance or large investments in conventional communication networks. Similarly, the combined use of aerial photogrammetry and mobile laser scanning ensures comprehensive coverage of the study area while minimizing data acquisition and processing times, enabling detailed and efficient documentation of the surveyed environment. Additionally, targeting points were placed at the sensor installation locations to ensure their correct spatial location within the photogrammetric model. These targets facilitated the precise identification of the sensors’ positions in the captured images, improving the integration of the data and enabling an accurate correlation between the photogrammetric information and the measurements recorded by the devices installed in the ground.

On top of that, the geospatial information and IoT monitoring data have been systematically complemented by the results obtained from geophysical prospecting conducted on the mining waste deposit. This comprehensive approach has enabled robust multimodal integration of diverse datasets, providing an effective characterization of the evaluated environment. By combining surface data derived from the geospatial techniques with subsurface information gathered through geophysical prospecting, this study achieves a holistic understanding of both the surface conditions and the underlying geological and structural features of the site. This integrated methodology enhances the accuracy and depth of the environmental assessment, facilitating future management and remediation efforts.

Continuous monitoring was prioritized because hazardous-waste deposits are subject to rapid and sometimes unpredictable changes triggered by external drivers such as extreme rainfall, groundwater table fluctuations, and progressive geotextile deterioration. These processes can lead to leachate migration or localized surface failure within short timeframes. If monitoring were carried out only every six or twelve months, such events would go unnoticed, resulting in delayed detection and potentially severe environmental consequences. By integrating real-time IoT sensing with geophysical data, the Digital Twin enables the early warning of anomalies through KPI-based alerts, enabling preventive interventions before significant damage occurs.

Furthermore, these technologies have opened up new opportunities for public participation and transparency in environmental restoration processes. By providing access to information on environmental quality and rehabilitation progress, public trust and collaboration among local communities, authorities, and other stakeholders are fostered. The active participation of citizens and community groups in surveillance and monitoring not only improves the effectiveness of the measures adopted but also generates new solutions and approaches that enrich the process. Access to real-time information also strengthens environmental education and public awareness, promoting a stronger commitment to sustainable management.

In addition to contributing to the safe management of hazardous materials present in the Park, this Digital Twin enables continuous assessment of progress in remediation efforts. These innovative tools not only support sustainable management practices but also improve decision-making processes and promote the preservation of the Park as a cultural and industrial heritage site in an environmentally responsible and technologically advanced manner.

Looking ahead, the implementation of artificial intelligence (AI)-driven analytics and advanced simulation models could further enhance the predictive capabilities of the developed Digital Twin. Additionally, fostering synergies with citizen science initiatives and educational institutions could promote a more inclusive, socially driven approach to environmental restoration. These developments would solidify the LTCP platform not only as a heritage management tool but also as a living laboratory for innovation, resilience, and community engagement.

One of the key advantages of the developed Digital Twin is its ability to simulate future scenarios based on historical sensor data and geophysical measurements. For instance, an increase in soil conductivity and moisture detected by the NPK sensors can be used to predict potential leachate migration, triggering preventive maintenance actions. Time-series analysis combined with machine learning techniques (e.g., ARIMA or Random Forest regressors) can be implemented to forecast parameter evolution under different environmental conditions. Preliminary tests demonstrated that predicted moisture variations matched the real measurements with a mean absolute error below 5%, validating the potential of the model for proactive risk management.

Although the system integrates several technologies, its implementation is cost-effective because it relies on affordable hardware and open-source software. Each LoRaWAN sensor node has a reduced price, and a single gateway can provide full coverage of the site. The use of TTN and Node-RED eliminates license fees, and the modular design enables stakeholders to begin with a minimal number of sensors and expand the system as needed. Considering the potential costs of remediation or structural repair if contamination events go undetected, the investment required for continuous monitoring is comparatively low, making this approach economically sustainable and scalable.

### Comparative Analysis with Similar Initiatives

The present subsection shows a comparative analysis of the present research study against similar initiatives. The objective is to contextualize the work within the broader landscape of related projects, highlighting both common approaches and distinctive contributions. By examining similarities and differences, the analysis aims to provide a deeper understanding of this study’s relevance, originality, and potential impact.

In this sense, comparable initiatives can be identified worldwide, providing a broader framework to contextualize the contribution of this study. For instance, in New Zealand, OceanaGold implemented a live Digital Twin of the Waihi Mine tailings storage facilities (TSFs), enabling continuous monitoring, advanced analysis, and governance support through integrated tools (iTwin, Leapfrog, or GeoStudio). This development illustrates the transition from reactive monitoring strategies to proactive management practices [67,68]. In Sweden, LKAB, together with Luleå University of Technology, maintains a Digital Twin of underground galleries generated from repeated LiDAR mapping (including robot-assisted surveys) to support routine inspections and safety decisions [69].

In addition, University of Oxford’s Digital Twins 4 Tailings Dams project uses multi-sensor satellite observations to build Digital Twin-based early warning for TSF stability [70]. In the case of Chile, continuous instrumentation and remote data acquisition have been deployed at facilities such as the Las Tórtolas TSF, and operators have reported real-time digital data collection workflows for tailings monitoring [71].

Taken together, the above projects demonstrate a clear trend toward integrating IoT-based sensing with 3D/4D modeling for proactive risk management in mining environments. The present study diverges from these approaches by (i) addressing a sealed hazardous-waste landfill located within an industrial heritage park, (ii) combining geophysical imaging techniques (GPR and UAV magnetometry) with surface–subsurface 3D models, and (iii) developing an immersive VR interface designed to support both environmental management and cultural valorization.

Regarding the geophysical integration for landfills, its use is also supported by prior studies. Multi-method investigations have combined GPR with magnetometry and/or resistivity to locate buried waste, delineate leachate, and assess structural conditions in landfills in North America and Europe [72,73]. Recent work further reports GPR-based assessment of contamination plumes and waste volumes, and UAV-based magnetometry has been validated to map ferromagnetic anomalies at waste sites [74,75].

Within this context, the present study’s contribution lies in establishing the spatial correlation of geophysical indicators with real-time IoT data inside a voxelized data model, subsequently presented through an interactive Digital Twin. Relative to existing benchmarks, the Lavrion DT integrates environmental monitoring and industrial heritage functions by unifying sensor-driven KPIs, geophysical evidence, and 3D/VR exploration into a single operational interface. Whereas most mining Digital Twins focus on operational TSFs or active underground workings, this case addresses a sealed hazardous-waste repository within a rehabilitated cultural park, simultaneously targeting safety concerns and public communication.

## 5. Conclusions

This study demonstrates the potential of Digital Twin technologies as a transformative tool for the integrated management, monitoring, and valorization of post-industrial environments, with a specific focus on the LTCP. By combining geospatial digitization, IoT sensor networks, geophysical diagnostics, and interactive visualization within a unified digital framework, the project delivers a comprehensive representation of both the surface and subsurface conditions of the site.

The developed Digital Twin enables real-time environmental monitoring, supports early detection of degradation risks, and enhances transparency and stakeholder engagement. The use of KPIs to trigger alerts based on sensor and geophysical data empowers proactive intervention and informed decision making. Furthermore, the interactive virtual tour offers a powerful communication and educational tool, bridging scientific knowledge with public accessibility.

The modular and scalable architecture of the proposed system enables its replication in other mining or environmentally degraded sites, making it a transferable model for smart remediation and heritage conservation. The integration of low-cost, low-power technologies (such as LoRaWAN-based sensors) ensures long-term sustainability, while the open-access platform promotes participatory governance and public awareness.

Looking toward the future, the integration of AI-based analytics, predictive simulations, and immersive technologies (VR/AR) will further increase the analytical and communicative potential of the platform. This work thus contributes not only to environmental restoration and heritage preservation but also to the broader goal of developing intelligent, inclusive, and resilient post-industrial territories in line with European sustainability objectives.

Although this research study is not conceived as a prescriptive guide, it provides a comprehensive methodological framework that can be adapted to other contaminated sites or post-mining areas. The workflow integrating IoT sensing, geophysical imaging, and 3D/VR modeling is described with enough detail to enable replication and scaling depending on the specific requirements and available resources of other projects.

## Figures and Tables

**Table 1:** General characteristics of LoRaWAN technology.

Technical Specifications	Values
Communication protocol	LoRaWAN (Long-Range Wide-Area Network)
Frequency	Depends on region: 868 MHz (Europe), 915 MHz (USA), and 433 MHz (some regions)
Programming interface	API based on AT commands or specific SDKs (LoRaWAN Stack)
Input/output	Varies according to module, typically UART, SPI, and I^2^C interfaces for connection to microcontrollers
Communication protocols	LoRaWAN, with support for MQTT, HTTP, and other protocols at the application layer
Energy supply	3.3–5 V DC (depending on the module); ultra-low power consumption in standby mode
Signal sensitivity	Up to −137 dBm at SF12 (high sensitivity to long distances)
Connexion topology	Star-of-Stars, with nodes communicating with gateways that relay data back to a network server

**Table 2:** Main characteristics of NPK-7in1 LoRaWAN Soil NPK EU868 sensor.

Parameter	Specification
Measurements	Nitrogen (N), phosphorus (P), potassium (K), soil moisture, soil temperature, electrical conductivity (EC), and salinity
Temperature Range	−30–70 °C
Moisture Range	0–100% (m^3^/m^3^)
Conductivity Range	0 to 2000 μS/cm
NPK range	0 to 1999 mg/kg (mg/L)
Measurement Accuracy	Temperature: ±0.2 °C; moisture: ±2% (m^3^/m^3^)
Resolution	Temperature: 0.1 °C; humidity: 0.1%; conductivity: 1 μS/cm; NPK: 1 mg/kg (mg/L)
Out Signal	RS485 (standard Modbus-RTU protocol)
Supply Voltage	6–24 V DC
Working Range	−30–70 °C
Stabilization Time	3 s after switching on
Response Time	<1 s
IP Rating	IP68 for the probe, suitable for long-term use buried in the ground
Battery	8500 mAh Li-SOCI2 battery, designed for up to 5 years of use
Wireless Connectivity	LoRaWAN 1.0.3 Class A technology, compatible with EU868 bands
Remote Configuration	Support for remote configuration via Bluetooth v5.1 and LoRaWAN, as well as OTA firmware updates
Applications	Intelligent agriculture, environmental soil monitoring

## Data Availability

The original contributions presented in this study are included in the article. Further inquiries can be directed to the corresponding author(s).
